# Colorectal Cancer Screening Program in Songjiang district, Shanghai between 2015 and 2017: Evaluation of participation rate and the associated factor

**DOI:** 10.1097/MD.0000000000029259

**Published:** 2022-08-12

**Authors:** Yiling Wu, Hiroaki Saito, Akihiko Ozaki, Tetsuya Tanimoto, Yonggen Jiang, Peng Yang, Jing Li, Zhiming Zhou, Xiuguo Zhu, Fei Lu, Yoshiaki Kanemoto, Tomohiro Kurokawa, Masaharu Tsubokura, Genming Zhao

**Affiliations:** a Songjiang Center for Disease Control and Prevention, Shanghai, China; b Department f Gastroenterology, Sendai Kousei Hospital, Miyagi, Japan; c Department of Radiation Health Management, Fukushima Medical University School of Medicine, Fukushima, Japan; d Department of Breast and Thyroid Surgery, Jyoban Hospital of Tokiwa Foundation, Fukushima, Japan; e Medical Governance Research Institute, Tokyo, Japan; f Department of Public Health, Zhongshan Street Community Health Service Center, Shanghai, China; g Department of Public Health, Sheshan Community Health Service Center, Shanghai, China; h Department of Public Health, Maogang Community Health Service Center, Shanghai, China; i Department of Public Health, Xinqiao Community Health Service Center, Shanghai, China; j Department of Surgery, Jyoban Hospital of Tokiwa Foundation, Fukushima, Japan; k School of Public Hsealth, Fudan University, Shanghai, China.

**Keywords:** colonic diseases, colorectal neoplasms, early detection of cancer, mass screening, risk factors

## Abstract

Little is known about the participation rate of newly implemented colorectal cancer (CRC) screening programs in China. Our goals were to identify factors associated with nonparticipation for CRC screening in Songjiang District, Shanghai.

We analyzed individuals included in an observational cohort study from 4 towns (Xin Qiao, She Shan, Mao Gang, and Zhong Shan) in Songjiang District. The participation rate was calculated for the CRC screening program based on a fecal immunochemical test and a risk assessment questionnaire between 2015 and 2017 inclusive.

Of the 27,130 individuals eligible for inclusion in this study, 20,863 (76.9%) participated in CRC screening at least once during 2015 and 2017. The factors linked with nonparticipation were; being male (odds ratio [OR] 0.87, 95% confidence interval [CI] 0.82–0.93, *P* < .01), unmarried (OR 0.71, 95% CI 0.64–0.80, *P* < .01), having a high education level (middle school, OR 0.82, 95% CI 0.74–0.90, *P* < .01, high school or above, OR 0.64, 95% CI 0.57–0.73, *P* < .01), absence of chronic disease (OR 0.90, 95% CI 0.85–0.96, *P* < .01), and living in 2 out of the 4 towns covered (Xin Qiao, OR 0.72, 95% CI 0.66–0.78, *P* < .01, Zhong Shan, OR 0.29, 95% CI 0.26–0.31, *P* < .01).

The current study revealed several associated factors with nonparticipation for the CRC screening in Songjiang district. These findings will help identify target populations that require an individualized approach to increase the participation rate.

## 1. Introduction

Health care screening programs are undertaken to help detect diseases preemptively in seemingly healthy people. There is a wide range of diseases and conditions to be screened for, including communicable diseases^[1,2]^ and noncommunicable diseases such as cancers.^[3,4]^ A public organization, such as a central or local government, often provides the screening tests, because screening programs can prevent or lower the prevalence of diseases in the population, help delay or stop the emergence of diseases in the future and facilitate the provision of early treatment via early diagnosis, improving the health and well-being of the population and reducing the social burden of the disease.^[5]^ In recent years, the role of cancer screening, with incidences of and deaths from cancers increasing, has become increasingly important worldwide.^[6]^

Colorectal cancer (CRC) is one of the leading causes of cancer deaths globally. It was estimated that there were >1.8 million new CRC cases worldwide in 2018,^[7]^ which was only behind lung cancer and breast cancer, while the number of deaths worldwide were also estimated to be relatively high at 880,000, second only to lung cancer. CRC tends to be more prevalent in countries with developed economies as it is associated with modern lifestyles, and its incidence is predicted to increase in the future as a result of population aging and global socioeconomic improvements.^[8]^ In continuing efforts to lower the overall global public health burden of CRC, CRC screening has been significantly expanded in recent years,^[9]^ and is now implemented virtually worldwide.

Among cancer screenings, CRC screening is one of the most established and effective. Most CRC progresses through adenoma-carcinoma, with an estimated interval of 10 to 15 years before CRC develops.^[10]^ Removal of polyps by colonoscopy can substantially reduce CRC incidence.^[11]^ Population-based CRC screening, therefore, can decrease the number of deaths from CRC and its mortality rate by detecting early-stage CRC or adenomas and offering curative treatment for them. The key indicators of the effectiveness of CRC screening are rates of participation, cancer detection, and comprehensive follow-up.^[12]^ As such, increasing the number of people who get screened is an essential goal in public health interventions.

In this context, CRC screening is provided in an inexpensive way in most countries, such as via fecal immunochemical tests (FITs) or fecal occult blood tests (FOBTs). Nevertheless, participation rates generally remain low (3%–69%), in both developing and developed countries.^[13–15]^ The factors affecting CRC screening rates differ among countries but those related to increased uptake include physician recommendations or a routine visit to a physician,^[16–18]^ knowledge about CRC,^[17]^ health care coverage,^[16]^ and family history of CRC.^[18]^ Thus, identifying reasons for nonparticipation in a specific population and finding ways to overcome them will also be key to ensuring future success of any CRC screening program.

In China, the burden of CRC is increasing, primarily due to a combination of Westernization of people’s diet and the aging population. With an age-standardized CRC incidence rate of 22.42 per 100,000 people and age-standardized CRC death rate of 10.10 per 100,000 in 2017, CRC ranked 11th in all causes of death in China, compared to 21st place in 1990.^[19]^ With an estimated newly diagnosed 521,490 cases, equivalent to about 28% of the global total, CRC was the second most common cancer in China in 2018, behind only lung cancer.^[20]^ The incidence of CRC patients in China is characterized by a regional difference as well. The coastal region has a higher age-adjusted mortality rate, and residents living in urban areas have a higher mortality rate than those living in the suburbs, and this trend is expected to continue.^[21]^ This can be explained by urbanization and the changing lifestyles that accompany it, along with the elevated economic power of residents. Therefore, it is necessary to take such factors and differences into consideration when making public health plans to tackle CRC in China.

Among Chinese cities, Shanghai is at the forefront of rapid economic development. And there have been several medical studies carried out in Songjiang District, southwest of Shanghai.^[22,23]^ In 2012, the Centers for Disease Control and Prevention (CDC) in Songjiang District started CRC screening for the residents. Although Songjiang District is located in a suburban area of Shanghai, it is undergoing rapid and diverse economic development, and conducting a study of cancer screening in such an area will provide important information and evidence for policy making. It should provide guidance for screening projects in other locations in China. The aims of this study were to analyze the CRC screening participation rate in Songjiang District using the data derived from a cohort study, and to identify factors associated with nonparticipation.

## 2. Materials and Methods

### 2.1. Study participants

This is a retrospective observational study that focused on a population enrolled in a cohort study to establish a database for noncommunicable diseases, which started in 2016. A detailed explanation of this cohort is described elsewhere,^[23–25]^ but its brief description is as follows; all residents of a randomly selected neighborhood community in the 4 towns in Songjiang district, Xin Qiao, She Shan, Mao Gang, and Zhong Shan, were considered as eligible. Residents were interviewed and their gender, age, occupation, health insurance status, medical history were collected and compiled in a database at the time of enrollment for the cohort.

### 2.2. CRC screening program

The CRC screening had been performed in Songjiang since 2012, with a 3-year period being designated as 1 round. The CRC screening period was from April to October in each year. All eligible residents in the cohort were asked to undergo FIT in the first year in each round. The target population of the CRC screening was adults aged 50 to 74 years. Each individual was assigned a unique identification number, which is common in the cohort study and CRC screening databases. By matching data from the cohort with data from the CRC screening, we could compare the details of those who participated from the cohort in CRC screening and those who did not. The first 3-year-round ran from 2012 to 2014 inclusive. In the present study, we analyzed the participation rate of the second round from 2015 to 2017. To investigate participation rates over round 2, we analyzed those who were 50 to 72 years old in 2015 and eligible for the second round.

### 2.3. Procedure of CRC screening in Songjiang

The screening consisted of primary screening and secondary screening. The primary screening included an FIT test and a face-to-face questionnaire for risk assessment. Subsequently, a secondary screening with a total colonoscopy was provided for participants found to be positive during the primary screening. In the primary screening process, participants were asked to visit respective community health service centers (CHCs) to complete a face-to-face questionnaire and collect 2 sample containers for the FIT test. Each participant was asked to return the containers within 48 hours after collection of their fecal material at home. The questionnaire, comprising 9 questions, including on anorectal symptoms, related diseases, and cancer history, was conducted by trained staff. The FIT test results were sent by mail and those with either a positive FIT result or positive risk assessment result were asked to attend the secondary screening in the same year.

### 2.4. Invitation of residents to the CRC screening

All eligible residents were encouraged to participate in the primary screening during the first year of each round through advertisements and through personal contact by district representatives. In 2015, all eligible residents were invited to take part in the CRC screening. In 2016 and 2017, those who tested positive in the previous year were invited to participate. The measures of contact varied; community centers in 2 towns, Mao Gang and She Shan, created contact lists for targeted people and individually called them on the telephone to encourage participation. Two other towns (Xin Qiao and Zhong Shan) created advertisements including flyers to encourage people to participate.

### 2.5. Statistical analysis

We defined CRC screening participation rates as the percentage of residents who participated in the CRC screening program, that is, those who had been screened at least once in a 3-year period (2015 to 2017) in the cohort population. Those with incomplete information obtained in the interviews at the enrollment of the cohort were excluded. Continuous variables were presented as median and interquartile range (IQR). The Mann-Whitney U test was used to compare continuous variables, and the chi-square test was used to compare categorical variables. Second, we constructed a univariate logistic regression model for participation in the program, using sex, age, working status (currently working or after retirement), marriage status (currently married or other), type of occupation, level of education, medical insurance status, chronic disease status, smoking status, and town of residence as covariates. Residents reported they had hypertension, coronary heart disease, hyperlipidemia, or diabetes were combined into ones as those with chronic diseases. Because there were few residents with university level education, we combined the education level category of high school with college or university for the regression analysis. We considered a *P* value of <.01 to be statistically significant and constructed a multivariate regression model for participation. Variable selection was performed with the backward elimination method. The analysis was performed using Statistical Package for the Social Science version 19 (IBM, Armonk, NY). The present study was performed in accordance with the Declaration of Helsinki and was approved by the Ethical Board at the School of Public Health of Fudan University (#2016-04-0586). The written informed consent to participate was obtained from all participants.

## 3. Results

In the cohort population, 27,130 people were eligible for the study (Table 1). This encompassed 5194 (19.1%) from Xin Qiao, 7889 (29.1%) from She Shan, 8760 (32.3%) from Mao Gang, and 5287 (19.5%) from Zhong Shan, respectively. The median age of the study population was 60 years (IQR: 55–66) and 15,788 (58.2%) were women. Of the total study population, 25,227 (93.0%) were married and 20,339 (75.0%) were retired. A total of 14,754 (54.8%) worked in companies and factories, with 3522 (13.0%) working in agriculture, 2480 (9.1%) freelance, 1457 (5.4%) unemployed, 684 (2.5%) in management of institutions or enterprises, and the remaining 4122 (15.2%) in other jobs. A total of 26,973 people (99.4%) had some kind of medical insurance. A total of 13,641 (50.3%) had at least 1 chronic disease, and 461 (1.7%) reported a personal history of cancer.

**Table 1:** Characteristics of target population.

Variables	Total (n = 27,130)
Age*, yrs, median (IQR)	60 (55–65)
Female, n (%)	15,788 (58.2)
Married, n (%)	25,227 (93)
Retired, n (%)	20,339 (75)
Occupation, n (%)	
Industrial enterprises	14,854 (54.8)
Agriculture	3533 (13.0)
Freelance	2480 (9.1)
Unemployed	1457 (5.4)
Organization/institutions†	684 (2.5)
Others	4122 (15.2)
Education, n (%)	
Illiterate	5128 (18.9)
Primary school	10,396 (38.3)
Middle school	9097 (33.5)
High school	2452 (9.0)
College/university	57 (0.21)
With medical insurance, n (%)	26,973 (99.4)
With chronic disease, n (%)	13,641 (50.3)
With cancer history, n (%)	461 (1.7)
With family’s cancer history, n (%)	35 (0.13)
Town, n (%)	
Xin Qiao	5194 (19.1)
She Shan	7889 (29.1)
Mao Gang	8760 (32.3)
Zhong Shan	5287 (19.5)

Of the 27,130 residents, 20,863 (76.9%) took an FIT test and the questionnaire at least once between 2015 and 2017; 13,167 (48.5%) did so once, 5138 (18.9%) did twice, and 2558 (9.4%) did 3 times. The median age of participants and nonparticipants were 61 years (IQR; 55–66) versus 59 years (IQR; 53–59) (*P* < .01), with females accounting for 58.9% versus 55.9% (*P* < .01) and those married making up 93.4% versus 91.8% (*P* < .01) (Table 2). The participation rates were 77.1% (4007) in Xin Qiao, 82.0% (6469) in She Shan, 83.6% (7324) in Mao Gang, and 57.9% (3063) in Zhong Shan (*P* < .01).

**Table 2:** Characteristics of participants and nonparticipants.

Variables	Participants (n = 20,863)	Nonparticipants (n = 6267)	*P* value†
Age*, yrs, median (IQR)	61 (55-66)	59 (53-59)	<.01
Female, n (%)	12,286 (58.9)	3502 (55.9)	<.01
Married, n (%)	19,476 (93.4)	5751 (91.8)	<.01
Retired, n (%)	15,773 (75.6)	4566 (72.9)	<.01
Occupation, n (%)			
Industrial enterprises	11,500 (55.12)	3354 (53.52)	<.01
Agriculture	2671 (12.8)	862 (13.75)	
Freelance	1958 (9.39)	522 (8.33)	
Unemployed	1109 (5.32)	348 (5.55)	
Organization/institutions‡	437 (2.09)	247 (3.94)	
Others	3188 (15.28)	934 (14.9)	
Education, n (%)			<.01
Illiterate	4133 (19.81)	995 (15.88)	
Primary school	8375 (40.14)	202 1 (32.25)	
Middle school	6678 (32.01)	2419 (38.6)	
High school	1661 (7.96)	791 (12.62)	
College/University	16 (0.08)	41 (0.65)	
With medical insurance, n (%)	20,751 (99.5)	6222 (99.3)	.097
With chronic diseases, n (%)	10,502 (49.31)	2944 (45.36)	<.01
With cancer history, n (%)	366 (1.7)	95 (1.5)	.20
With family’s cancer history, n (%)	32 (0.15)	3 (0.05)	.06
Town, n (%)			
Xin Qiao	4007 (19.2)	1187 (21)	<.01
She Shan	6469 (31.0)	1420 (21.93)	
Mao Gang	7324 (35.1)	1436 (22.8)	
Zhong Shan	3063 (14.7)	2224 (34.27)	

Univariable logistic regression analysis revealed 8 variables as statistically relevant factors for participation (*P* < .01); age, sex, marriage status, working status, type of occupation, education level, status of chronic disease, and town of residence. Multivariable logistic regression analysis with these 8 variables (Table 3) showed that being less likely to participate was associated mainly with males (OR 0.87, 95% CI 0.82–0.93, *P* < .01), those not currently married (OR 0.71, 95% CI 0.64–0.80, *P* < .01), having a higher education level (middle school, OR 0.82, 95% CI 0.74–0.90, *P* < .01, high school or above, OR 0.64, 95% CI 0.57–0.73, *P* < .01), or those with a chronic disease (OR 0.90, 95% CI 0.85–0.96, *P* < .01), and with respect to place of residence, (Xin Qiao, OR 0.72, 95% CI 0.66–0.78, *P* < .01, Zhong Shan, OR 0.29, 95% CI 0.26–0.31, *P* < .01).

**Table 3:** Factors associated with participation rate; multivariable logistic regression analysis.

	OR	95% CI	*P* value
Sex			
Women	Reference	–	–
Men	0.874	0.821–0.931	<.01
Age			
50–59 yr	0.873	0.813–0.938	<.01
60–69 yr	Reference	60–69	–
70–72 yr	0.866	0.776–0.967	.011
Marital status			
Married	Reference	–	–
Others	0.711	0.636–0.795	<.01
Employment status			
Retired	Reference	–	–
Working	0.999	0.922–1.083	.982
Occupation			
Industrial enterprises	Reference	–	–
Agriculture	0.968	0.885–1.060	.482
Freelance	1.108	0.995–1.234	.062
Unemployed	0.982	0.862–1.120	.788
Organization/institutions*	0.713	0.602–0.845	<.01
Others	1.003	0.921–1.094	.939
Education			
Illiteracy	Reference	–	–
Primary school	1.103	1.009–1.207	.031
Middle school	0.818	0.742–0.901	<.01
High school and above	0.644	0.569–0.729	<.01
With chronic diseases			
Yes	Reference	–	–
No	0.904	0.852–0.959	<.01
Town			
Mao Gang	Reference	–	–
She Shan	0.927	0.853–1.006	.069
Xin Qiao	0.717	0.656–0.783	<.01
Zhong Shan	0.286	0.264–0.309	<.01

## 4. Discussion

As the second round of CRC screening held in Songjiang had a high participation rate (76.9%), the mobilization of the community seemed to have been relatively successful. However, there remains a sizable percentage of nonparticipation, and various factors were detected to be associated with this. Our findings should inform future screening programs in ways that should improve their performance and outcomes.

The participation rate identified in CRC screening in Songjiang was high compared to those reported in Western countries and other Asian countries, although the rates are not easily comparable as our screening covered a 3-year period. Guidelines for screening for CRC in Europe and the United States recommend screening for FIT and FOBT every 1 to 2 years for people at normal risk.^[26,27]^ In the United States, CRC screening in the general population was reported to be 62.4% in 2015^[28]^ and 82.7% in a population in which screening for other cancers was also organized.^[29]^ Japan has reported rates of 17.0% to 41.4%^[30–33]^ and in China, rates have been as low as 17.3% to 35.2%.^[13,34]^ One of the factors that contributed to the high participation rate in the current study would be the low financial burden of the program. The CRC screening program in Songjiang District costs 5.6 to 7 RMB (US$0.7 to 0.9) per initial FIT inspection, but the program is offered at no cost to residents. Subsequent colonoscopies were also arranged to be covered by health insurance.^[35]^ The characteristics of the population will also have affected the results of the study. Our study targeted the population involved in the cohort study, who would have had a higher health-consciousness due to the prior regular exposure.

The current study is unique in the respect that we used a cohort so that we could identify factors associated with residents’ nonparticipation. Previous studies have shown that men, younger age, and nonmarried individuals are associated with lower CRC screening participation, and the results of the present study were consistent with these findings.^[36,37]^ However, there were a couple of notable observations that should be specifically discussed. First, a presence of chronic disease was associated with a participation in the CRC screening in the current study. A plausible reason is that, in regular hospital visits, physicians in charge may have recommended an uptake of CRC screening, given that such interventions have been reported to improve the participation rate in CRC screening.^[38,39]^ Second, there was an inverse relationships between educational levels and participation rate. Previously, a high level of education and awareness of the importance of CRC screening were reported to be associated with a higher participation rates in the United States and in other Asian countries, and the our findings contradict this.^[13,38]^ However, given that a similar phenomenon was observed about compliance with colonoscopy screening in Shanghai,^[35]^ there may have been some specific local mechanisms contributing as causal factors, an important topic that should be explored in future research.

It is well known that there are regional-level differences in CRC screening participation,^[40,41]^ and the current study also showed this trend. When the cohort was further divided into subregions, all 4 districts had high screening uptake rates, but the differences in screening participation rates among districts were statistically evident; residents in Xin Qiao or Zhong Shan were statistically less likely to participate in CRC screening compared to those in Mao Gang. Given that it has been reported that urban status and size of community are associated with CRC screening participation rates,^[42]^ an undetermined demographic element in each town may have affected CRC participation rates. In addition, it is notable that approaches adopted by community centers were different between the 4 towns. One example was the way in which local residents were contacted; community centers in Mao Gang and She Shan created contact lists for targeted people and individually called them on the telephone to encourage participation, while Xin Qiao and Zhong Shan just created flyers to encourage people to participate. However, this does not explain the difference in participation rate found between Xin Qiao and Zhong Shan. As such, an in-depth analysis should be conducted to more comprehensively evaluate the differences of CRC participation rates among the 4 towns.

### 4.1. Clinical implications and future perspectives

In this study, it was reassuring that the participation rate for CRC screening was high. To further enhance the participation rate among the local residents, it would be useful to undertake interventions focusing on improving characteristics relating to a low participation rate, such as an individualization of promotional methods. Given that several factors showed a unique association with a participation rate in our study, there would be a significant value in undertaking similar analyses in local public health teams, instead of just relying on aggregated evidence from previous screening programs.

In addition, this study should provide important insights to guide future studies about CRC screening programs in the Songjiang District. Specifically, a further exploration of the association between locations of local residents (town) and participation rate are important, given that they may be related to recruiting methods or other communication adopted by local health centers. Consequently, clarifications of the underlying reasons would easily lead to an increased participation rate in the towns demonstrating a low participation rate. We believe that these approaches would also contribute to an accumulation of evidence relating to CRC screening programs globally.

### 4.2. Limitations

The limitations of the study include that there was a 1-year gap in the database. The cohort was enrolled in 2016 and the CRC screening started in 2015, so there may be people who lived in areas not eligible for CRC screening in 2015 but who were enrolled in the 2016 cohort. Although the actual participation rate might be underestimated, we estimated the 1-year gap had little impact on this study because the population growth of the 4 towns included in the cohort over the previous year has been around only 1.2%. The current study included several diverse factors in the analysis; however, some known barriers to obtaining CRC screening, such as income level^[43]^ and accessibility,^[13]^ were not analyzed.

## 5. Conclusion

The current study analyzed some key factors affecting participation in CRC screening conducted in Shanghai’s Songjiang District. There were differences in the participation rate of residents among the 4 towns covered. Clarification and better and more comprehensive understanding of the actual status of CRC screening in each region is necessary in order to further increase the participation rate.

## Author contributions

G.Z. was the guarantor; Y.W., H.S., A.O., and T.T. designed the study and drafted the initial manuscript; Y.W. participated in the acquisition, analysis of the data; Y.J., P.Y., J.L., Z.Z., X.Z., F.L., Y.K., T.K., M.T., G.Z. revised the article critically for important intellectual content.

## Acknowledgments

The authors would like to express our gratitude to all the individuals who participated in, or staff who were involved in, the colorectal cancer screening in Songjiang district.

## Footnotes

* Age in 2015. People aged 50–72 in 2015 were eligible.

† Working as manager of institutions.

* Age in 2015. People aged 50–72 in 2015 were eligible.

† The Student t test was used to compare continuous variables, and the chi-square test was used to compare category variables.

‡ Working as manager of institutions.

* Working as a manager of institutions.
